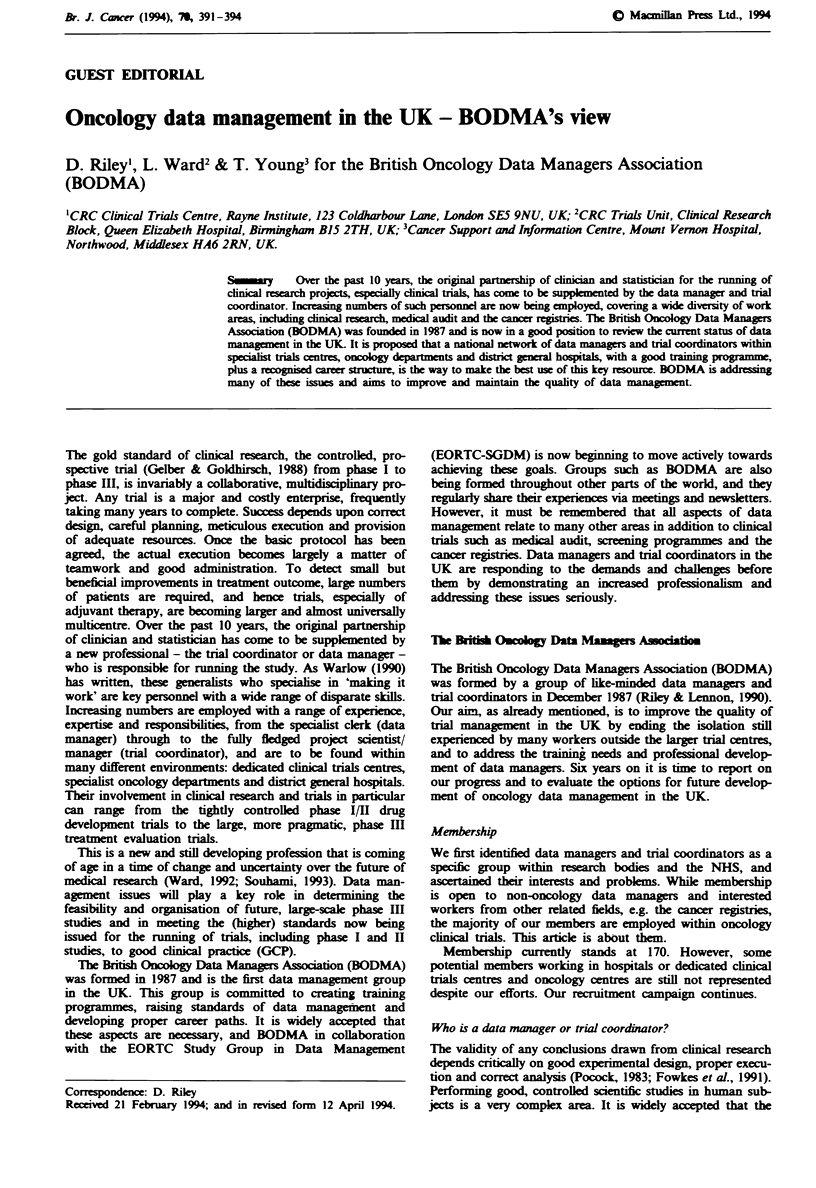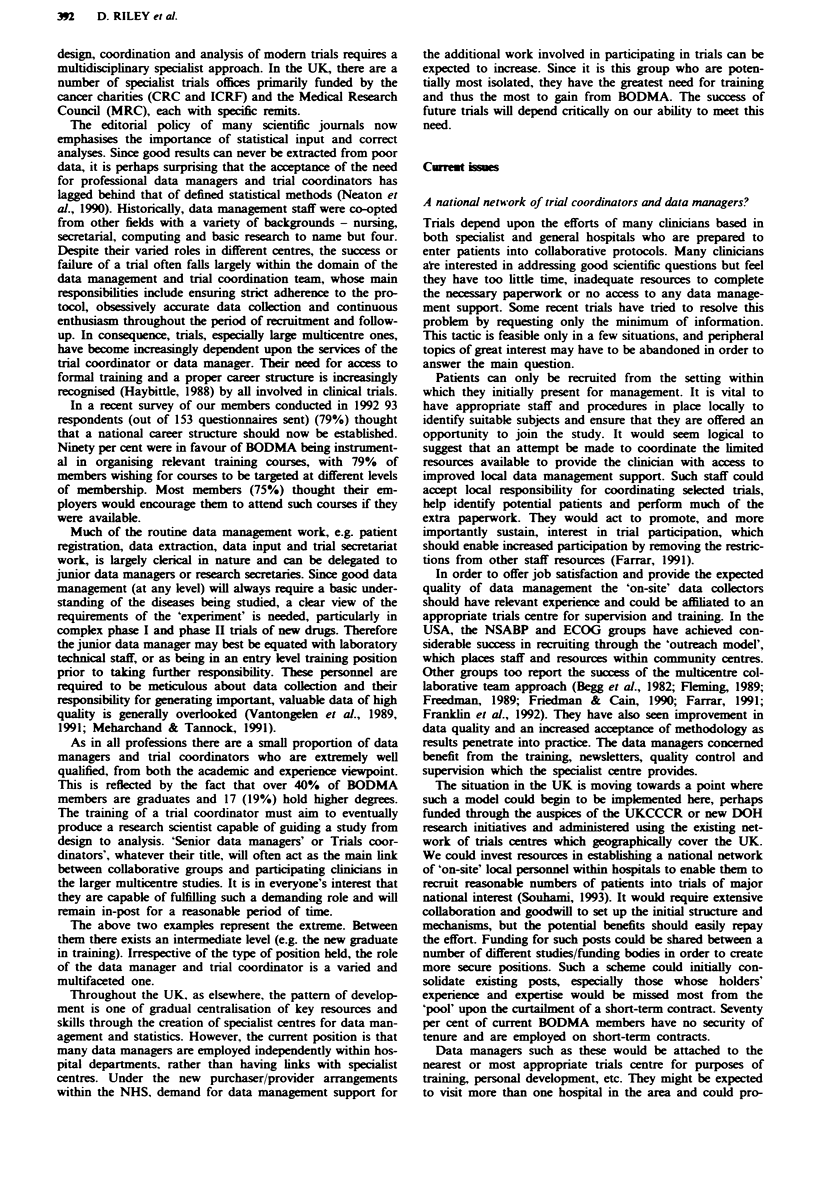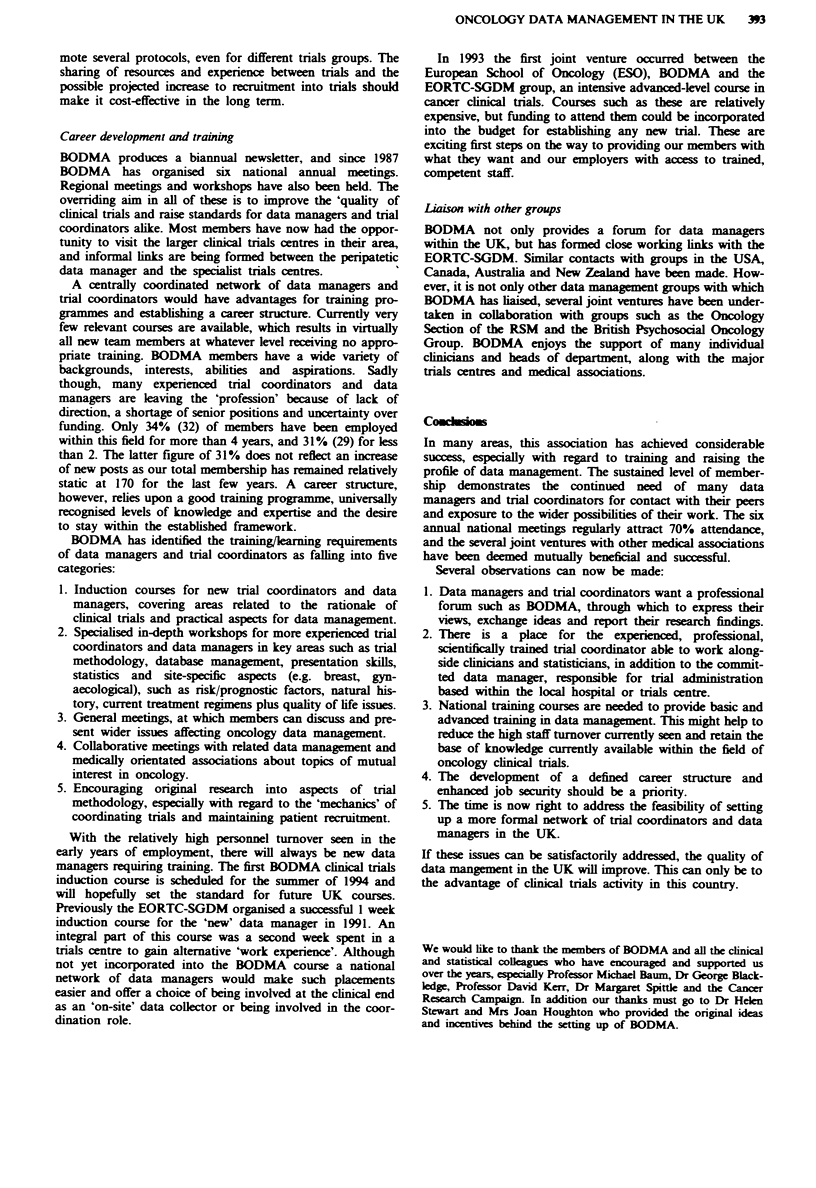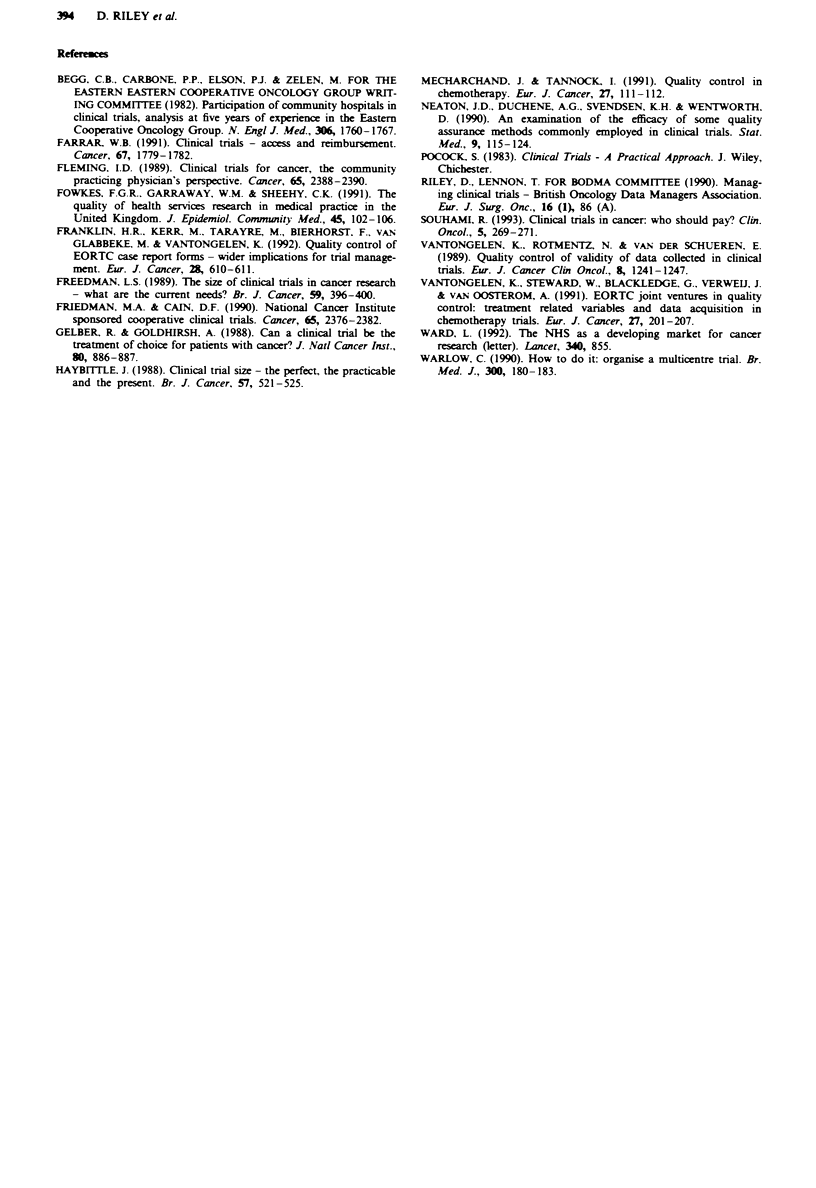# Oncology data management in the UK--BODMA's view. British Oncology Data Managers Association.

**DOI:** 10.1038/bjc.1994.314

**Published:** 1994-09

**Authors:** D. Riley, L. Ward, T. Young

## Abstract

Over the past 10 years, the original partnership of clinician and statistician for the running of clinical research projects, especially clinical trials, has come to be supplemented by the data manager and trial coordinator. Increasing numbers of such personnel are now being employed, covering a wide diversity of work areas, including clinical research, medical audit and the cancer registries. The British Oncology Data Managers Association (BODMA) was founded in 1987 and is now in a good position to review the current status of data management in the UK. It is proposed that a national network of data managers and trial coordinators within specialist trials centres, oncology departments and district general hospitals, with a good training programme, plus a recognised career structure, is the way to make the best use of this key resource. BODMA is addressing many of these issues and aims to improve and maintain the quality of data management.


© Macmillan Press Ltd., 1994

GUEST EDITORIAL

### Oncology data management in the UK – BODMA's view

D. Riley[1], L. Ward[2] & T. Young[3] for the British Oncology Data Managers Association (BODMA)

[1]*CRC Clinical Trials Centre, Rayne Institute, 123 Coldharbour Lane, London SE5 9NU, UK;* [2]*CRC Trials Unit, Clinical Research Block, Queen Elizabeth Hospital, Birmingham B15 2TH, UK;* [3]*Cancer Support and Information Centre, Mount Vernon Hospital, Northwood, Middlesex HA6 2RN, UK.*

**Summary** Over the past 10 years, the original partnership of clinician and statistician for the running of clinical research projects, especially clinical trials, has come to be supplemented by the data manager and trial coordinator. Increasing numbers of such personnel are now being employed, covering a wide diversity of work areas, including clinical research, medical audit and the cancer registries. The British Oncology Data Managers Association (BODMA) was founded in 1987 and is now in a good position to review the current status of data management in the UK. It is proposed that a national network of data managers and trial coordinators within specialist trials centres, oncology departments and district general hospitals, with a good training programme, plus a recognised career structure, is the way to make the best use of this key resource. BODMA is addressing many of these issues and aims to improve and maintain the quality of data management.

The gold standard of clinical research, the controlled, prospective trial (Gelber & Goldhirsch, 1988) from phase I to phase III, is invariably a collaborative, multidisciplinary project. Any trial is a major and costly enterprise, frequently taking many years to complete. Success depends upon correct design, careful planning, meticulous execution and provision of adequate resources. Once the basic protocol has been agreed, the actual execution becomes largely a matter of teamwork and good administration. To detect small but beneficial improvements in treatment outcome, large numbers of patients are required, and hence trials, especially of adjuvant therapy, are becoming larger and almost universally multicentre. Over the past 10 years, the original partnership of clinician and statistician has come to be supplemented by a new professional – the trial coordinator or data manager – who is responsible for running the study. As Warlow (1990) has written, these generalists who specialise in 'making it work' are key personnel with a wide range of disparate skills. Increasing numbers are employed with a range of experience, expertise and responsibilities, from the specialist clerk (data manager) through to the fully fledged project scientist/manager (trial coordinator), and are to be found within many different environments: dedicated clinical trials centres, specialist oncology departments and district general hospitals. Their involvement in clinical research and trials in particular can range from the tightly controlled phase I/II drug development trials to the large, more pragmatic, phase III treatment evaluation trials.

This is a new and still developing profession that is coming of age in a time of change and uncertainty over the future of medical research (Ward, 1992; Souhami, 1993). Data management issues will play a key role in determining the feasibility and organisation of future, large-scale phase III studies and in meeting the (higher) standards now being issued for the running of trials, including phase I and II studies, to good clinical practice (GCP).

The British Oncology Data Managers Association (BODMA) was formed in 1987 and is the first data management group in the UK. This group is committed to creating training programmes, raising standards of data management and developing proper career paths. It is widely accepted that these aspects are necessary, and BODMA in collaboration with the EORTC Study Group in Data Management (EORTC-SGDM) is now beginning to move actively towards achieving these goals. Groups such as BODMA are also being formed throughout other parts of the world, and they regularly share their experiences via meetings and newsletters. However, it must be remembered that all aspects of data management relate to many other areas in addition to clinical trials such as medical audit, screening programmes and the cancer registries. Data managers and trial coordinators in the UK are responding to the demands and challenges before them by demonstrating an increased professionalism and addressing these issues seriously.

### The British Oncology Data Managers Association

The British Oncology Data Managers Association (BODMA) was formed by a group of like-minded data managers and trial coordinators in December 1987 (Riley & Lennon, 1990). Our aim, as already mentioned, is to improve the quality of trial management in the UK by ending the isolation still experienced by many workers outside the larger trial centres, and to address the training needs and professional development of data managers. Six years on it is time to report on our progress and to evaluate the options for future development of oncology data management in the UK.

### *Membership*

We first identified data managers and trial coordinators as a specific group within research bodies and the NHS, and ascertained their interests and problems. While membership is open to non-oncology data managers and interested workers from other related fields, e.g. the cancer registries, the majority of our members are employed within oncology clinical trials. This article is about them.

Membership currently stands at 170. However, some potential members working in hospitals or dedicated clinical trials centres and oncology centres are still not represented despite our efforts. Our recruitment campaign continues.

### *Who is a data manager or trial coordinator?*

The validity of any conclusions drawn from clinical research depends critically on good experimental design, proper execution and correct analysis (Pocock, 1983; Fowkes *et al.*, 1991). Performing good, controlled scientific studies in human subjects is a very complex area. It is widely accepted that the

Correspondence: D. Riley
Received 21 February 1994; and in revised form 12 April 1994.

design, coordination and analysis of modern trials requires a multidisciplinary specialist approach. In the UK, there are a number of specialist trials offices primarily funded by the cancer charities (CRC and ICRF) and the Medical Research Council (MRC), each with specific remits.

The editorial policy of many scientific journals now emphasises the importance of statistical input and correct analyses. Since good results can never be extracted from poor data, it is perhaps surprising that the acceptance of the need for professional data managers and trial coordinators has lagged behind that of defined statistical methods (Neaton *et al.*, 1990). Historically, data management staff were co-opted from other fields with a variety of backgrounds – nursing, secretarial, computing and basic research to name but four. Despite their varied roles in different centres, the success or failure of a trial often falls largely within the domain of the data management and trial coordination team, whose main responsibilities include ensuring strict adherence to the protocol, obsessively accurate data collection and continuous enthusiasm throughout the period of recruitment and follow-up. In consequence, trials, especially large multicentre ones, have become increasingly dependent upon the services of the trial coordinator or data manager. Their need for access to formal training and a proper career structure is increasingly recognised (Haybittle, 1988) by all involved in clinical trials.

In a recent survey of our members conducted in 1992 93 respondents (out of 153 questionnaires sent) (79%) thought that a national career structure should now be established. Ninety per cent were in favour of BODMA being instrumental in organising relevant training courses, with 79% of members wishing for courses to be targeted at different levels of membership. Most members (75%) thought their employers would encourage them to attend such courses if they were available.

Much of the routine data management work, e.g. patient registration, data extraction, data input and trial secretariat work, is largely clerical in nature and can be delegated to junior data managers or research secretaries. Since good data management (at any level) will always require a basic understanding of the diseases being studied, a clear view of the requirements of the 'experiment' is needed, particularly in complex phase I and phase II trials of new drugs. Therefore the junior data manager may best be equated with laboratory technical staff, or as being in an entry level training position prior to taking further responsibility. These personnel are required to be meticulous about data collection and their responsibility for generating important, valuable data of high quality is generally overlooked (Vantongelen *et al.*, 1989, 1991; Meharchand & Tannock, 1991).

As in all professions there are a small proportion of data managers and trial coordinators who are extremely well qualified, from both the academic and experience viewpoint. This is reflected by the fact that over 40% of BODMA members are graduates and 17 (19%) hold higher degrees. The training of a trial coordinator must aim to eventually produce a research scientist capable of guiding a study from design to analysis. 'Senior data managers' or Trials coordinators', whatever their title, will often act as the main link between collaborative groups and participating clinicians in the larger multicentre studies. It is in everyone's interest that they are capable of fulfilling such a demanding role and will remain in-post for a reasonable period of time.

The above two examples represent the extreme. Between them there exists an intermediate level (e.g. the new graduate in training). Irrespective of the type of position held, the role of the data manager and trial coordinator is a varied and multifaceted one.

Throughout the UK, as elsewhere, the pattern of development is one of gradual centralisation of key resources and skills through the creation of specialist centres for data management and statistics. However, the current position is that many data managers are employed independently within hospital departments, rather than having links with specialist centres. Under the new purchaser/provider arrangements within the NHS, demand for data management support for the additional work involved in participating in trials can be expected to increase. Since it is this group who are potentially most isolated, they have the greatest need for training and thus the most to gain from BODMA. The success of future trials will depend critically on our ability to meet this need.

### Current issues

### *A national network of trial coordinators and data managers?*

Trials depend upon the efforts of many clinicians based in both specialist and general hospitals who are prepared to enter patients into collaborative protocols. Many clinicians are interested in addressing good scientific questions but feel they have too little time, inadequate resources to complete the necessary paperwork or no access to any data management support. Some recent trials have tried to resolve this problem by requesting only the minimum of information. This tactic is feasible only in a few situations, and peripheral topics of great interest may have to be abandoned in order to answer the main question.

Patients can only be recruited from the setting within which they initially present for management. It is vital to have appropriate staff and procedures in place locally to identify suitable subjects and ensure that they are offered an opportunity to join the study. It would seem logical to suggest that an attempt be made to coordinate the limited resources available to provide the clinician with access to improved local data management support. Such staff could accept local responsibility for coordinating selected trials, help identify potential patients and perform much of the extra paperwork. They would act to promote, and more importantly sustain, interest in trial participation, which should enable increased participation by removing the restrictions from other staff resources (Farrar, 1991).

In order to offer job satisfaction and provide the expected quality of data management the 'on-site' data collectors should have relevant experience and could be affiliated to an appropriate trials centre for supervision and training. In the USA, the NSABP and ECOG groups have achieved considerable success in recruiting through the 'outreach model', which places staff and resources within community centres. Other groups too report the success of the multicentre collaborative team approach (Begg *et al.*, 1982; Fleming, 1989; Freedman, 1989; Friedman & Cain, 1990; Farrar, 1991; Franklin *et al.*, 1992). They have also seen improvement in data quality and an increased acceptance of methodology as results penetrate into practice. The data managers concerned benefit from the training, newsletters, quality control and supervision which the specialist centre provides.

The situation in the UK is moving towards a point where such a model could begin to be implemented here, perhaps funded through the auspices of the UKCCCR or new DOH research initiatives and administered using the existing network of trials centres which geographically cover the UK. We could invest resources in establishing a national network of 'on-site' local personnel within hospitals to enable them to recruit reasonable numbers of patients into trials of major national interest (Souhami, 1993). It would require extensive collaboration and goodwill to set up the initial structure and mechanisms, but the potential benefits should easily repay the effort. Funding for such posts could be shared between a number of different studies/funding bodies in order to create more secure positions. Such a scheme could initially consolidate existing posts, especially those whose holders' experience and expertise would be missed most from the 'pool' upon the curtailment of a short-term contract. Seventy per cent of current BODMA members have no security of tenure and are employed on short-term contracts.

Data managers such as these would be attached to the nearest or most appropriate trials centre for purposes of training, personal development, etc. They might be expected to visit more than one hospital in the area and could promote several protocols, even for different trials groups. The sharing of resources and experience between trials and the possible projected increase to recruitment into trials should make it cost-effective in the long term.

### *Career development and training*

BODMA produces a biannual newsletter, and since 1987 BODMA has organised six national annual meetings. Regional meetings and workshops have also been held. The overriding aim in all of these is to improve the 'quality of clinical trials and raise standards for data managers and trial coordinators alike. Most members have now had the opportunity to visit the larger clinical trials centres in their area, and informal links are being formed between the peripatetic data manager and the specialist trials centres.

A centrally coordinated network of data managers and trial coordinators would have advantages for training programmes and establishing a career structure. Currently very few relevant courses are available, which results in virtually all new team members at whatever level receiving no appropriate training. BODMA members have a wide variety of backgrounds, interests, abilities and aspirations. Sadly though, many experienced trial coordinators and data managers are leaving the 'profession' because of lack of direction, a shortage of senior positions and uncertainty over funding. Only 34% (32) of members have been employed within this field for more than 4 years, and 31% (29) for less than 2. The latter figure of 31% does not reflect an increase of new posts as our total membership has remained relatively static at 170 for the last few years. A career structure, however, relies upon a good training programme, universally recognised levels of knowledge and expertise and the desire to stay within the established framework.

BODMA has identified the training/learning requirements of data managers and trial coordinators as falling into five categories:

1. Induction courses for new trial coordinators and data managers, covering areas related to the rationale of clinical trials and practical aspects for data management.
2. Specialised in-depth workshops for more experienced trial coordinators and data managers in key areas such as trial methodology, database management, presentation skills, statistics and site-specific aspects (e.g. breast, gynaecological), such as risk/prognostic factors, natural history, current treatment regimens plus quality of life issues.
3. General meetings, at which members can discuss and present wider issues affecting oncology data management.
4. Collaborative meetings with related data management and medically orientated associations about topics of mutual interest in oncology.
5. Encouraging original research into aspects of trial methodology, especially with regard to the 'mechanics' of coordinating trials and maintaining patient recruitment.

With the relatively high personnel turnover seen in the early years of employment, there will always be new data managers requiring training. The first BODMA clinical trials induction course is scheduled for the summer of 1994 and will hopefully set the standard for future UK courses. Previously the EORTC-SGDM organised a successful 1 week induction course for the 'new' data manager in 1991. An integral part of this course was a second week spent in a trials centre to gain alternative 'work experience'. Although not yet incorporated into the BODMA course a national network of data managers would make such placements easier and offer a choice of being involved at the clinical end as an 'on-site' data collector or being involved in the coordination role.

In 1993 the first joint venture occurred between the European School of Oncology (ESO), BODMA and the EORTC-SGDM group, an intensive advanced-level course in cancer clinical trials. Courses such as these are relatively expensive, but funding to attend them could be incorporated into the budget for establishing any new trial. These are exciting first steps on the way to providing our members with what they want and our employers with access to trained, competent staff.

### *Liaison with other groups*

BODMA not only provides a forum for data managers within the UK, but has formed close working links with the EORTC-SGDM. Similar contacts with groups in the USA, Canada, Australia and New Zealand have been made. However, it is not only other data management groups with which BODMA has liaised, several joint ventures have been undertaken in collaboration with groups such as the Oncology Section of the RSM and the British Psychosocial Oncology Group. BODMA enjoys the support of many individual clinicians and heads of department, along with the major trials centres and medical associations.

### Conclusions

In many areas, this association has achieved considerable success, especially with regard to training and raising the profile of data management. The sustained level of membership demonstrates the continued need of many data managers and trial coordinators for contact with their peers and exposure to the wider possibilities of their work. The six annual national meetings regularly attract 70% attendance, and the several joint ventures with other medical associations have been deemed mutually beneficial and successful.

Several observations can now be made:

1. Data managers and trial coordinators want a professional forum such as BODMA, through which to express their views, exchange ideas and report their research findings.
2. There is a place for the experienced, professional, scientifically trained trial coordinator able to work alongside clinicians and statisticians, in addition to the committed data manager, responsible for trial administration based within the local hospital or trials centre.
3. National training courses are needed to provide basic and advanced training in data management. This might help to reduce the high staff turnover currently seen and retain the base of knowledge currently available within the field of oncology clinical trials.
4. The development of a defined career structure and enhanced job security should be a priority.
5. The time is now right to address the feasibility of setting up a more formal network of trial coordinators and data managers in the UK.

If these issues can be satisfactorily addressed, the quality of data mangement in the UK will improve. This can only be to the advantage of clinical trials activity in this country.

We would like to thank the members of BODMA and all the clinical and statistical colleagues who have encouraged and supported us over the years, especially Professor Michael Baum, Dr George Blackledge, Professor David Kerr, Dr Margaret Spittle and the Cancer Research Campaign. In addition our thanks must go to Dr Helen Stewart and Mrs Joan Houghton who provided the original ideas and incentives behind the setting up of BODMA.

### References

BEGG, C.B., CARBONE, P.P., ELSON, P.J. & ZELEN, M. FOR THE EASTERN EASTERN COOPERATIVE ONCOLOGY GROUP WRITING COMMITTEE (1982). Participation of community hospitals in clinical trials, analysis at five years of experience in the Eastern Cooperative Oncology Group. *N. Engl J. Med.*, **306**, 1760-1767.

FARRAR, W.B. (1991). Clinical trials - access and reimbursement. *Cancer*, **67**, 1779-1782.

FLEMING, I.D. (1989). Clinical trials for cancer, the community practicing physician's perspective. *Cancer*, **65**, 2388-2390.

FOWKES, F.G.R., GARRAWAY, W.M. & SHEEHY, C.K. (1991). The quality of health services research in medical practice in the United Kingdom. *J. Epidemiol. Community Med.*, **45**, 102-106.

FRANKLIN, H.R., KERR, M., TARAYRE, M., BIERHORST, F., VAN GLABBEKE, M. & VANTONGELEN, K. (1992). Quality control of EORTC case report forms - wider implications for trial management. *Eur. J. Cancer*, **28**, 610-611.

FREEDMAN, L.S. (1989). The size of clinical trials in cancer research - what are the current needs? *Br. J. Cancer*, **59**, 396-400.

FRIEDMAN, M.A. & CAIN, D.F. (1990). National Cancer Institute sponsored cooperative clinical trials. *Cancer*, **65**, 2376-2382.

GELBER, R. & GOLDHIRSH, A. (1988). Can a clinical trial be the treatment of choice for patients with cancer? *J. Natl Cancer Inst.*, **80**, 886-887.

HAYBITTLE, J. (1988). Clinical trial size - the perfect, the practicable and the present. *Br. J. Cancer*, **57**, 521-525.

MECHARCHAND, J. & TANNOCK, I. (1991). Quality control in chemotherapy. *Eur. J. Cancer*, **27**, 111-112.

NEATON, J.D., DUCHENE, A.G., SVENDSEN, K.H. & WENTWORTH, D. (1990). An examination of the efficacy of some quality assurance methods commonly employed in clinical trials. *Stat. Med.*, **9**, 115-124.

POCOCK, S. (1983). *Clinical Trials - A Practical Approach*. J. Wiley, Chichester.

RILEY, D., LENNON, T. FOR BODMA COMMITTEE (1990). Managing clinical trials - British Oncology Data Managers Association. *Eur. J. Surg. Onc.*, **16 (1)**, 86 (A).

SOUHAMI, R. (1993). Clinical trials in cancer: who should pay? *Clin. Oncol.*, **5**, 269-271.

VANTONGELEN, K., ROTMENTZ, N. & VAN DER SCHUEREN, E. (1989). Quality control of validity of data collected in clinical trials. *Eur. J. Cancer Clin Oncol.*, **8**, 1241-1247.

VANTONGELEN, K., STEWARD, W., BLACKLEDGE, G., VERWEIJ, J. & VAN OOSTEROM, A. (1991). EORTC joint ventures in quality control: treatment related variables and data acquisition in chemotherapy trials. *Eur. J. Cancer*, **27**, 201-207.

WARD, L. (1992). The NHS as a developing market for cancer research (letter). *Lancet*, **340**, 855.

WARLOW, C. (1990). How to do it: organise a multicentre trial. *Br. Med. J.*, **300**, 180-183.